# Efficacy of a Low-FODMAP Diet for Coeliac Patients with Persistent IBS-like Symptoms despite a Gluten-Free Diet: A Systematic Review

**DOI:** 10.3390/nu16071094

**Published:** 2024-04-08

**Authors:** Francesca Lusetti, Annalisa Schiepatti, Davide Scalvini, Stiliano Maimaris, Federico Biagi

**Affiliations:** 1 Department of Internal Medicine and Therapeutics, University of Pavia, 27100 Pavia, Italy; 2 Istituti Clinici Scientifici Maugeri, Gastroenterology Unit of IRCCS Pavia Institute, University of Pavia, 27100 Pavia, Italy; 3 PhD Course in Experimental Medicine, University of Pavia, 27100 Pavia, Italy

**Keywords:** celiac disease, gluten-free diet, persistent symptoms, FODMAP, irritable bowel syndrome

## Abstract

**Background:** Persistent symptoms in coeliac disease (CD) can be due to not only poor gluten-free diet (GFD) adherence and complications of CD, but also functional gastrointestinal disorders such as irritable bowel syndrome (IBS). Although the role of a low fermentable oligo-, di-, and monosaccharides and polyols (FODMAP) diet is well-established in IBS, little data are available on its role in coeliac patients with persistent IBS-like symptoms despite a GFD. **Methods:** We systematically reviewed the literature in accordance with the PRISMA guidelines for studies evaluating the role of FODMAPs and/or a low-FODMAP diet in coeliac patients with persistent symptoms. PubMed and Embase were searched from inception to 16 January 2024 for eligible full-text papers. The study protocol was registered on Open Science Framework. **Results:** A total of 239 records were identified, and six papers were included. Of these, four were interventional studies comparing a low-FODMAP GFD to a regular GFD for persistent symptoms in 115 total coeliac patients (two randomized controlled trials and two open-label studies). A low-FODMAP GFD for a minimum of 4 weeks was significantly more effective than a regular GFD in reducing symptoms (*p* < 0.05 in 3/4 studies). Dietary FODMAP content of a conventional GFD was significantly lower than that of non-coeliac patients on a gluten-containing diet (both *p* < 0.05), especially regarding high-FODMAP grain products. However, coeliac patients consumed more servings of fruits/vegetables high in FODMAP. No relationship between FODMAP intake and persistence of symptoms was reported. **Conclusions:** A low-FODMAP diet may be beneficial for uncomplicated celiac patients with persistent IBS-like symptoms despite strict adherence to a GFD.

## 1. Introduction

Coeliac disease (CD) is a common immune-mediated enteropathy triggered by dietary gluten in genetically susceptible individuals, affecting around 1% of the general population and characterized by a heterogeneous clinical picture [1,2,3,4]. Diagnosis of CD in adults is based on villous atrophy and positive celiac-specific serology, including tissue transglutaminase and endomysial antibodies [1,2,3,4]. Although a strict lifelong gluten-free diet (GFD) is the mainstay for the treatment of CD, leading to the resolution of clinical symptoms and histological lesions in the vast majority of patients, [1,2,3,5] persistence of gastrointestinal symptoms despite a GFD is a relevant clinical scenario, occurring in up to 30–40% of coeliac patients [6,7,8,9,10,11,12]. Ongoing symptoms in treated coeliac patients are associated with a significant social and psychological burden, leading to a reduced quality of life (QOL) and increased healthcare expenditure [11,12,13].

Poor adherence to a GFD, either voluntarily or inadvertently, is a major cause of ongoing symptoms in coeliac patients [6,7,8,9,10,11,12], and, although rare, life-threatening conditions, such as refractory CD, lymphoma and other malignant complications of CD should be excluded [6,8,9,10,14,15]. Apart from these aetiologies, it has been shown that up to 50% of coeliac patients may experience ongoing chronic functional gastrointestinal symptoms that are compatible with functional gastrointestinal disorders (FGID) despite being on a GFD [6,7,10,11,12].

Irritable bowel syndrome (IBS) is a common chronic functional disorder with an estimated prevalence of 4–11% worldwide [16], characterized by symptoms such as abdominal pain, diarrhoea, constipation, bloating, which often overlap with symptoms of CD [16,17,18,19]. Abdominal and psychological symptoms of IBS are associated with a reduced QOL, increased healthcare utilization, and reduced productivity [16,17,18,19,20].

In the last few years, great attention has been devoted to dietary interventions and lifestyle modifications as therapeutic options for patients suffering from IBS; in particular, a short-term trial with a diet low in fermentable oligo-, di-, and monosaccharides and polyols (FODMAP) or a short-term GFD can be considered [17,18,19,20,21,22]. Some types of FODMAPs, such as fructose, lead to increased gastrointestinal water secretion, while others, such as fructans, are not fully digested in the small intestine and instead undergo fermentation by bacteria in the colon, resulting in the production of gas. These processes have the potential to trigger or worsen symptoms like abdominal pain, bloating, flatulence, and diarrhoea in individuals with IBS [21].

Although currently, there are no specific recommendations for the treatment of coeliac patients experiencing persistent symptoms despite a GFD, the possibility of dietary interventions in addition to a GFD for persistent IBS-like symptoms could be considered, but following a low-FODMAP diet in addition to a GFD may be challenging. In this regard, the dietitian can play a pivotal role in creating a personalized diet to relieve persistent symptoms and rebalancing the usual GFD, which, according to the literature, is often nutritionally inadequate [23].

To date, little is known about the possible role of a low-FODMAP diet in coeliac patients with persistent functional symptoms despite a GFD. The aim of this study is to provide a systematic literature review on this topic and evaluate the clinical efficacy and feasibility of this dietary intervention for coeliac patients with persistent symptoms despite a GFD.

## 2. Methods

### 2.1. Literature Search Details

A systematic review of the literature was conducted in accordance with the PRISMA 2020 Guidelines [24]. The systematic review protocol was prospectively registered on Open Science Framework (https://www.doi.org/10.17605/OSF.IO/GWEJ7, accessed on 1 June 2023). PubMed and Embase were searched from the database inception to 20 January 2023 for papers reporting on the efficacy of a low-FODMAP diet as a treatment in patients with CD. The search was subsequently updated on 16 January 2024. Search terms for CD and FODMAP or a low-FODMAP diet were used. The bibliographies of selected studies and reviews were also hand-searched to identify any other relevant studies not identified by our database search. No language restrictions were used in the search. The exact search strings used for PubMed and Embase were as follows: PubMed: (celiac disease[mesh] OR coeliac disease OR celiac disease) AND (diets, FODMAP[mesh] OR fodmap) and Embase: (‘coeliac disease’/exp OR ‘coeliac disease’ OR ‘celiac disease’/exp OR ‘celiac disease’) AND (‘FODMAP diet’ OR ‘fodmap’).

### 2.2. Eligibility Criteria

Studies meeting all the following criteria were considered for inclusion: (1) full-text papers on adult (≥18 years old) or pediatric (<18 years old) patients with a confirmed diagnosis of CD and on a GFD and (2) clinical trials or observational studies reporting on the efficacy of a low-FODMAP diet or any possible relationship between FODMAP dietary intake and symptoms, as well as studies reporting on the FODMAP content of a conventional GFD.

Studies reporting on the role of a low-FODMAP diet only in diseases other than CD or the efficacy of only other dietary treatments in addition to a low-FODMAP diet were excluded. Review papers, conference abstracts, and case reports were also excluded.

### 2.3. Data Extraction

Two reviewers (FL and DS) independently screened titles and abstracts of records retrieved by the literature search to identify potentially relevant studies. Disagreements were resolved by discussion and/or with the assistance of other reviewers (AS and SM). Potentially relevant studies underwent full-text screening for eligibility by at least two reviewers. For each eligible paper, data were extracted on study characteristics, study population, and study outcomes. For studies where multiple analyses were conducted with adjustment for different variables, the most adjusted-for analysis was preferred. For studies reporting outcome measures separately for different groups of patients, these were also extracted separately for each group. Contacting study authors was considered in case of studies not reporting important data in the original paper.

### 2.4. Outcomes

We aimed to evaluate the following outcomes: (1) the efficacy of a low-FODMAP diet in coeliac patients with gastrointestinal symptoms despite a GFD; (2) whether dietary FODMAP intake is related to the persistence of symptoms in coeliac patients on a regular GFD; and (3) an evaluation of the dietary FODMAP content of a conventional GFD.

### 2.5. Study Quality and Risk of Bias

Study quality and risk of bias were assessed independently for each study by two reviewers. The Cochrane RoB 2.0 tool was used to assess the risk of bias for randomised controlled trials. This tool evaluates the following sources of bias: (1) bias arising from the randomization process; (2) bias due to deviations from intended interventions; (3) bias due to missing outcome data; (4) bias in measurement of the outcome; and (5) bias in selection of the reported result. The overall risk of bias was graded as low-risk (low-risk for all domains), some concerns (some concerns in at least one domain), or high risk (high-risk in at least one domain). Non-randomized interventional studies were evaluated using the ROBINS-I tool, which evaluates: (1) bias due to confounding, (2) bias in the selection of participants for the study, (3) bias in the classification of intervention, (4) bias due to deviations from intended interventions, (5) bias due to missing data, (6) bias in the measurement of outcomes, and (7) bias in the selection of the reported result. The overall risk of bias was categorised as low (low risk in all domains), moderate (low or moderate risk in all domains), serious (serious risk in at least one domain), or critical (critical risk in at least one domain). For observational studies, the Newcastle–Ottawa scale for cohort studies and an adapted version for cross-sectional studies were used. These scales evaluate: (1) selection of study groups, (2) comparability, and (3) ascertainment of the outcome of interest.

Studies were evaluated as being as at low risk of bias if they scored within 1 point from the maximum score, moderate risk if they scored 2 points below the maximum, and high risk if they scored 3 or more points below the maximum score. Disagreements were resolved by discussion and/or consultation with a third reviewer.

## 3. Results

### 3.1. Characteristics of Included Studies

**Figure 1:**
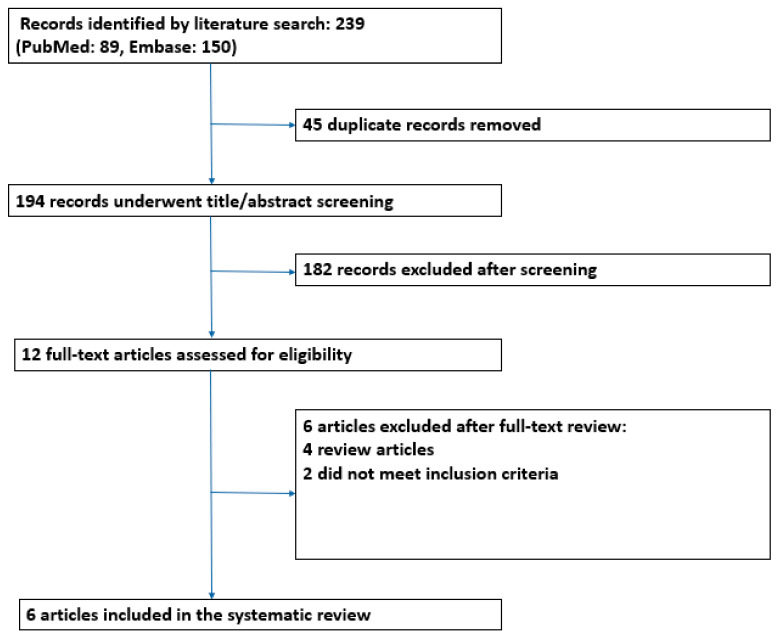
Study flowchart showing papers excluded and included for the systematic review.

As shown in Figure 1, our literature search identified 239 records, of which six were eligible for inclusion after full-text review. The characteristics of the included studies are summarized in Table 1. Four were interventional studies (two randomized controlled trials—RCT [25,26], two open-label prospective interventional studies [27,28]), and two were observational studies (one retrospective cohort study [29] and one cross-sectional study [30]). Studies excluded after full-text review and the reasons for exclusion are reported in Supplementary Materials. Overall, the risk of bias among the included studies was high.

### 3.2. Efficacy of a Low-FODMAP Diet Intervention in Celiac Patients with Persistent Symptoms

As shown in Table 1, four interventional studies (two RCT and two open-label interventional studies) investigated the efficacy of a low-FODMAP GFD compared to a regular GFD for persistent symptoms in CD. All the studies included coeliac patients in remission (serologic remission for two studies [26,27] and serologic and mucosal recovery for the other two studies [25,28]), with IBS-like symptoms despite good adherence to a GFD.

Overall, 176 celiac patients were included and, of these, 115 underwent a low-FODMAP GFD (all adults, including 86 females). All four studies found that a low-FODMAP GFD was effective at reducing symptoms. In particular, the efficacy of the intervention was assessed with the Gastrointestinal Symptom Rating Scale (GSRS)–IBS version in two studies [25,28], whereas in the other two studies were assessed with a visual analogic scale (VAS) [26] and IBS severity scoring system (IBS-SSS) [27]. Both Van Megen et al. and Trott et al. found that following a low-FODMAP GFD for 4 weeks significantly reduced symptoms measured by GSRS-IBS compared to a regular GFD in the first study (*p* interaction < 0.001) [25] and compared to baseline symptoms in the second study (*p* = 0.007) [28]. On the other hand, Roncoroni et al. found that 21 days of a low-FODMAP GFD significantly reduced abdominal pain compared to a regular GFD (*p* < 0.01) [26]. Finally, in the study by Testa et al., gastrointestinal symptoms, evaluated with the IBS-SSS questionnaire, improved after 1 and 3 months of a low-FODMAP diet in all patients [27]. As shown in Table 1, this study included patients with IBS, IBD, and CD and found similar rates of improvement between these groups (*p* = NS).

### 3.3. Effect of a Low-FODMAP Diet on Quality of Life and Psychological and General Well-Being

The effect of a low-FODMAP diet in improving QOL and psychological morbidity in coeliac patients was evaluated by two of the included studies (see Table 1) [26,27]. More precisely, an RCT by Roncoroni et al. highlighted that psychological symptoms and general well-being significantly improved in the intervention group after 21 days of a low-FODMAP diet compared to the control group [26]. Specifically, the Symptom Checklist-90-R (SCL-90) questionnaire and the Short Form 36 HeFalth Survey (SF-36) questionnaire were assessed at baseline and after 21 days of intervention in both groups to evaluate the presence and severity of symptoms of mental distress and the quality of life of the patients. The low-FODMAP GFD group showed a remarkable decrease in most SCL-90 scores; in particular, the global SCL-90 score was significantly reduced compared to the regular GFD group at day 21 (*p* < 0.0003). Conversely, there was no significant reduction in SCL-90 scores observed in the regular GFD group.

Similarly, a dietary interventional prospective study by Testa et al. showed an improvement of QOL after 3 months of a low-FODMAP diet in patients with IBS, inflammatory bowel disease in remission, and CD, although this was not statistically significant [27]. QOL, as in the aforementioned study by Roncoroni et al., was assessed with the Short Form-36 (SF-36) questionnaire at baseline and after 1 and 3 months of dietary intervention. The authors found a significant improvement from T0 to T3 in most of the domains of the questionnaire, even if a direct comparison among the three groups did not demonstrate any statistically significant result (*p* = NS).

### 3.4. Relationship of FODMAP Intake with Persistent Symptoms in Coeliac Patients on a Gluten-Free Diet

Two studies investigated the relationship between FODMAP intake and persistent symptoms in coeliac patients on a GFD [29,30]. Both studies, one in adults and the other in children, found that coeliac patients on a GFD had an overall lower FODMAP intake than healthy controls. They also found no relationship between FODMAP intake and gastrointestinal symptoms [29,30]. Both reported a higher consumption of cereals/grains and sweets with high FODMAP content in non-coeliac controls, whereas in coeliac patients, higher consumption of fruit and vegetables with a high FODMAP content was reported [29,30].

### 3.5. Risk of Bias Evaluation

Details on risk of bias evaluation for each domain and overall risk of bias judgment for each included study are available in Supplementary Materials (Supplementary Table S2). Risk of bias evaluation revealed that almost all the included studies were at high risk of bias [30]. More precisely, the two randomised controlled trials were both found to be at high risk of bias due to possible deviations from the intended intervention, as assessed using the Cochrane RoB 2.0 tool. Major factors contributing to risk of bias in these two trials included the use of a per-protocol analysis rather than an intention-to-treat analysis [25,26] and lack of blinding in the trial by van Megen et al. [25].

With regard to the two prospective interventional studies by Testa et al. [27] and Trott et al. [28], both were found to be at critical risk of bias, as assessed by the ROBINS-I tool. Significant limitations included a lack of control of possible confounding factors, possible risk of bias in the measurement of outcomes, and deviation from the intended intervention. Moreover, neither of these two studies incorporated a control group; so, the role of placebo on the study results cannot be excluded [27,28]. Finally, the two observational studies investigating FODMAP intake of coeliac patients on a GFD were considered, respectively, at medium risk of bias [30] and at high risk of bias [29]. Specifically, the study by Cyrkot et al. [30] lost one point in both the selection and comparability domains, while the study by Roncoroni et al. [29] lost two points in the comparability domain and one point in both the selection and outcome domains.

## 4. Discussion

This systematic review has summarized the current evidence about the efficacy of a 4–12-week course of a low-FODMAP diet as a treatment for patients with CD experiencing persistent functional IBS-like symptoms despite a GFD.

The problem of persistent symptoms despite a GFD is a common clinical scenario, affecting up to 30–40% of coeliac patients and can be due to different underlying aetiologies [6,7,8,9,10,11,12]. Inadequate adherence to a GFD, due either to voluntary or involuntary transgressions, has been reported as the leading cause for persistent symptoms in coeliac patients [6,7,8,9]. Despite this, rates of adherence to a GFD can vary widely among coeliac patients [5,10,31] as dietary adherence can be influenced by economic, social, and psychological factors, as well as proper instruction on how to correctly follow a GFD [5,10,32,33,34].

Life-threatening complications of CD such as refractory CD, intestinal lymphomas, and small-bowel adenocarcinomas can severely worsen the prognosis of coeliac patients [10,15,35,36]. However, although these complications must be carefully excluded in patients with persistent or recurrent symptoms despite a GFD, these are fortunately rare [10,15,35,36].

After poor adherence to a GFD, complications of CD, and other organic disorders are excluded, it has been shown that symptoms due to functional gastrointestinal disorders such as IBS, oesophageal reflux disease, bloating and dyspepsia are among the most common underlying aetiologies for persistent symptoms in coeliac patients [6,7,8,9,10,11,12,37]. Moreover, in these patients with persistent functional gastrointestinal symptoms, it is possible that some of them may also be super-sensitive to minimal quantities of gluten ingested inadvertently [6]. However, this aspect is in need of further clarification as the findings of a previous study by our group did not support the role of minimal quantities of gluten inadvertently ingested in triggering symptoms in coeliac patients with evidence of mucosal healing who had been instructed on how to follow a strict GFD [38].

Our systematic review of the literature has shown that overall, a short course of a low-FODMAP diet for 4–12 weeks was effective in managing adult coeliac patients with persistent IBS-like symptoms despite a GFD. A significant improvement in secondary outcomes, including quality of life, psychological, and overall well-being, was also shown [26,27]. On the other hand, despite the apparent efficacy of a low-FODMAP diet in treating IBS-like symptoms in coeliac patients, the dietary FODMAP intake of coeliac patients on a GFD was found to be lower overall than in non-coeliac controls in observational studies [29,30]. It is, however, noteworthy that in coeliac patients on a GFD, the FODMAP intake derived from cereals consumption has decreased, whereas the FODMAP intake derived from vegetables and fruit has increased, which reflects the change in dietary composition necessarily originating from a GFD [29,30].

Although a low-FODMAP diet for managing persistent symptoms in coeliac patients can be in contrast to an apparently lower FODMAP intake of patients on a GFD, there are a number of factors that should be considered. First, although, overall, patients on a GFD may have a lower FODMAP intake, there appears to be a subset of celiac patients with persistent IBS-like symptoms who may nevertheless benefit from dietary rebalancing. Even if the small number of studies and their small sample size do not allow to identify the phenotype of patients who may benefit from a low-FODMAP diet, based on our clinical experience and the available literature [39,40,41], it is likely that young adult females with anxiety and IBS-like symptoms with a hypervigilant approach to the diet may represent the target population for this dietary intervention. Second, the lower FODMAP intake of celiac patients was primarily due to a reduced intake of cereals/grains despite a higher intake of fruits and vegetables high in FODMAP. This is likely to be due to the exclusions of many cereals as part of following a GFD [41].

Based on the results of this review, a short course of low-FODMAP diet can be considered in coeliac patients with persistent IBS-like symptoms after the exclusion of other causes for persistent symptoms, including poor GFD adherence, complications of CD, and other organic disorders. However, this intervention should be guided by expert dietitians in order to avoid an overly restrictive diet, nutritional deficiencies, or the risk of a low-quality diet high in processed foods, which may increase the risk of developing cardio-metabolic disorders [42,43,44,45]. Moreover, following a low-FODMAP diet in addition to a GFD may be psychologically and economically demanding [46], thus possibly representing a major barrier to an effective treatment. This is a further reason for which the decision to start a low-FODMAP diet in addition to a GFD should be strictly based on expert dietitian advice, with the provision of a personalised dietary programme to ensure the maintenance of a high-quality balanced diet [47,48].

Current guidelines on the management of patients with functional gastrointestinal disorders suggest a short course of a low-FODMAP diet for effectively managing symptoms and improving the quality of life in these patients [17,18,19,20,21,22]. Moreover, the role of a low-FODMAP diet has also been investigated in RCTs in patients with organic diseases such as inflammatory bowel disease, with promising results [49,50]. A single-blind RCT by Cox et al. [49] enrolled 52 patients with quiescent Crohn’s disease or ulcerative colitis and persistent gut symptoms and randomly assigned the patients to follow a diet low in FODMAPs (n = 27) or a control diet (n = 25) for 4 weeks. At the end of the study, a greater proportion of patients reported relief of gastrointestinal symptoms following the low-FODMAP diet (14/27, 52%) than the control diet (4/25, 16%, *p* = 0.007). Patients in the low-FODMAP diet group also had higher health-related quality of life scores (81.9 ± 1.2) than patients on the control diet (78.3 ± 1.2, *p* = 0.042) [49].

An Italian study by Bodini et al. [50] also found an improvement of faecal inflammatory markers and quality of life in patients with mainly quiescent disease. This study enrolled fifty-five patients with inflammatory bowel disease in remission or with mild disease activity (as assessed by a Mayo score <6 in patients with UC and a Harvey–Bradshaw Index (HBI) < 8 in patients with CD) and randomized the patients for a 6-week low-FODMAP diet or standard diet. Disease activity, faecal calprotectin, and disease-specific quality of life (IBD-Q) were assessed at baseline and at the end of dietary intervention. Interestingly, after the dietary intervention, median HBI decreased in the low-FODMAP diet group (4; IQR, 3–5 versus 3; IQR, 2–3; *p* = 0.024) but not in the standard diet (3; IQR, 3–3 versus 3; IQR, 2–4), whereas Mayo scores were lower in the low-FODMAP diet group and unmodified in the standard diet group. Median calprotectin also decreased significantly in the low-FODMAP diet group (from 76.6 mg/kg; IQR, 50–286.3 to 50 mg/kg; IQR, 50.6–81; *p* = 0.004) but not in the standard diet group. Lastly, the authors also observed a borderline significant increase in median IBD-Q in the low-FODMAP diet group (*p* = 0.05) with no change in the standard diet group [50].

Although the results of our systematic review of the literature suggest that a low-FODMAP diet may be beneficial in coeliac patients with persistent symptoms and demonstrate the relevant implications of this for clinical practice, several limitations must be acknowledged. These include the relatively small number of included studies, their small sample size, the heterogeneity of the study populations, and the lack of standardization of treatment duration and outcome measures. The risk of bias evaluation of the studies included in our systematic review also revealed that overall, most were at high risk of bias in one or more domains. Moreover, only two randomized controlled studies were available for inclusion. Due to the limited quantity and quality of evidence and the heterogeneity of the included studies, it was not possible to conduct a quantitative meta-analysis of the data to reach more definitive conclusions about the efficacy of a low-FODMAP dietary intervention in celiac patients with persistent IBS-like symptoms.

In conclusion, a short course of a low-FODMAP gluten-free diet based on expert dietitian advice may be an effective intervention in coeliac patients with persistent IBS-like symptoms after other causes have been systematically excluded.

## Figures and Tables

**Table 1:** Summary of studies evaluating the efficacy of a FODMAP diet in patients with celiac disease.

Study	Country	Study Design	Enrolment Criteria	Intervention	Patients, N	Primary Outcome	Primary Outcome Results	Secondary Outcomes	Secondary Outcome Results
Roncoroni 2018 [29]	Italy	Retrospective cohort study	Adult patients with CD on a GFD	None	104 CD 91 healthy controls	Dietary FODMAP intake	FODMAP intake was lower in celiac patients on a GFD (*p* < 0.001)	Prevalence of IBS and FGIDs	No significant difference in the prevalence of IBS and FGIDs in CD vs. controls (*p:* NS)
Van Megen 2022 [25]	Norway	Open-label parallel-group RCT	Adult celiac patients on a GFD for ≥12 months with serologic and mucosal remission and persistent symptoms (GSRS-IBS ≥ 30)	Low-FODMAP GFD vs. usual GFD for 4 weeks	70 CD (34 in the intervention group and 36 in the control group)	Improvement of symptoms (reduction in GSRS-IBS ≥ 7) by week 4	GSRS-IBS was significantly lower at week 4 in the intervention group (MD −10.8, 95% CI −6.8 to −14.8) (*p* interaction < 0.001)	CSI score, CFQ score, and FODMAP intake at week 4	All secondary outcome measures were significantly lower in the intervention group (CSI score: *p* = 0.003; CFQ: *p* = 0.02; FODMAP intake *p* < 0.001)
Roncoroni 2018 [26]	Italy	RCT	Adult patients with CD on a GFD for ≥12 months with serologic remission and IBS-like symptoms according to the Rome III criteria and a global well-being score assessed by a VAS < 4	Low-FODMAP GFD vs. usual GFD for 21 days	50 CD (25 in the intervention group 25 in the control group)	Improvement of gastrointestinal symptoms and general well-being (assessed by a VAS), psychological symptoms (SCL-90), and QOL (SF-36) after 21 days	Reduced global SCL-90 index (*p* < 0.0003) in the intervention group. Lower VAS for abdominal pain (*p* < 0.01) and higher VAS for faecal consistency (*p* < 0.09) in the intervention group. General well-being improved more in the intervention group (*p* = 0.03)	-	-
Testa 2018 [27]	Italy	Dietetic interventional prospective study	Adult patients with CD on a GFD for ≥12 months with serologic remission and IBS-like symptoms according to the Rome III criteria	Low-FODMAP diet (LOW-FODMAP GFD for the CD group) for 3 months	127 pts: 56 with IBS, 30 with IBD in clinical remission, and 41 with CD	Improvement of gastrointestinal symptoms (IBS-SSS) after 1 and 3 months	Gastrointestinal symptoms improved after 1 and 3 months in all patients, with no significant difference between the groups (*p* = NS)	Improvement of QOL (SF-36)	No difference between the 3 groups in terms of response to diet (*p* = NS), but there was a clinical improvement after 3 months for most of the questionnaire’s domains
Trott 2021 [28]	UK	Open-label prospective interventional pilot study	Adult patients with CD on a GFD for ≥24 months in mucosal remission and IBS-like symptoms according to the Rome III criteria	Low-FODMAP diet (no comparator) for 4 weeks	15 CD	Improvement of symptoms (evaluated with GSRS-IBS) after a minimum of 4 weeks of an adjuvant low-FODMAP diet	Global relief of gut symptoms reported by 8/15 patients (53% *p* = 0.007), with significant reductions in abdominal pain (*p* < 0.01), distension (*p* < 0.02), and flatulence (*p* < 0.01).	-	-
Cyrkot 2021 [30]	Canada	Cross-sectional study	Children aged 5–18 years with biopsy-proven CD on GFD	None	46 CD 46 non-celiac mild chronic gastrointestinal complaints (GIC) 46 healthy controls (HC)	Evaluation of the association between FODMAP consumption and gastrointestinal symptoms (PedsQLTM GI Symptom Scale ([GSS]), diet quality (Canadian Healthy Eating Index (HEI-C)), and health-related quality of life (PedsQLTM 4.0 Generic Core Scales)	CD children consumed fewer foods high in FODMAPs compared to GIC and HC (*p* < 0.0001). FODMAP intake was not related to GSS in CD children (*p* > 0.05) but positively associated with child health-related quality of life (*p* < 0.05). FODMAP intake from fruits and vegetables was positively associated with diet adequacy and total diet quality in CD children (*p* < 0.05).	-	-

CD: celiac disease; GFD: gluten-free diet; FODMAP: fermentable oligo-,di-, and monosaccharides and polyols; IBS: irritable bowel syndrome; FGIDs: functional gastrointestinal disorders; RCT: randomized controlled trial; GSRS: Gastrointestinal Symptom Rating Scale; CSI score: Celiac Symptom Index score; CFQ score: Chalder Fatigue Questionnaire; VAS: visual analogic scale; SCL-90: Symptom Checklist-90-R; QOL: quality of life; SF-36: Short Form (36) Health Survey questionnaires; IBS-SSS: Irritable Bowel Syndrome Severity Scoring System; PedsQLTM GI Symptom Scale ([GSS]), PedsQLTM 4.0 Generic Core Scales.

## Supplementary Materials

The following supporting information can be downloaded at: https://www.mdpi.com/article/10.3390/nu16071094/s1, Table S1: Papers excluded after full-text review; Table S2: Risk of bias assessment.
