# Supplementary material for: Efficacy of a Low-FODMAP Diet for Coeliac Patients with Persistent IBS-like Symptoms despite a Gluten-Free Diet: A Systematic Review
Source: Nutrients. 2024 Apr 8;16(7):1094. doi: 10.3390/nu16071094 (PMC11013587; doi:10.3390/nu16071094)
Supplement: Supplementary file 1 [file nutrients-16-01094-s001.zip › nutrients-2885377-supplementary.pdf]

Literature search details

PubMed and Embase were searched from database inception to 20-Jan-2023 for papers reporting on the efficacy of a low-FODMAP diet as a treatment in patients with coeliac disease. The search was subsequently repeated and updated on 16-Jan-2024. No language restrictions were used in the search. The exact search strings used for PubMed and Embase are as follows.

PubMed: (celiac disease[mesh] OR coeliac disease OR celiac disease) AND (diets, FODMAP[mesh] OR fodmap)

Embase: ('coeliac disease'/exp OR 'coeliac disease' OR 'celiac disease'/exp OR 'celiac disease') AND ('FODMAP diet' OR 'fodmap')

Supplementary Table S1. Papers excluded after full-text review.

Paper	Reason for exclusion
Muir JG, Varney JE, Ajamian M, et al. Gluten-free and low-FODMAP sourdoughs for patients with coeliac disease and irritable bowel syndrome: A clinical perspective. Int J Food Microbiol 2019;290:237-246. doi: 10.1016/j.ijfoodmicro.2018.10.016.	Article is a review
Yoosuf S, Makharia GK. Evolving Therapy for Celiac Disease. Front Pediatr 2019;7:193. doi: 10.3389/fped.2019.00193.	Article is a review
Reddel S, Putignani L, Del Chierico F. The Impact of Low-FODMAPs, Gluten-Free, and Ketogenic Diets on Gut Microbiota Modulation in Pathological Conditions. Nutrients 2019;11:373. doi: 10.3390/nu11020373.	Article is a review
Bascuñán KA, Elli L, Pellegrini N, et al. Impact of FODMAP Content Restrictions on the Quality of Diet for Patients with Celiac Disease on a Gluten-Free Diet. Nutrients 2019;11:2220. doi: 10.3390/nu11092220.	Article discusses effect of a low-FODMAP GFD on nutritional adequacy but not on persistent symptoms and therefore did not meet inclusion criteria
Patel PK, Tanpowpong P, Sriaroon P, et al. Nonallergic Diseases Associated With Foods. J Allergy Clin Immunol Pract 2023:S2213-2198(23)01058-9. doi: 10.1016/j.jaip.2023.09.027. Epub ahead of print.	Article is a review
Herfindal AM, van Megen F, Gilde MKO, et al. Effects of a low FODMAP diet on gut microbiota in individuals with treated coeliac disease having persistent gastrointestinal symptoms - a randomised controlled trial. Br J Nutr. 2023;130:2061-2075. doi: 10.1017/S0007114523001253.	Article discusses effects of a low-FODMAP GFD on gut microbiota but does not investigate clinical efficacy on persistent symptoms and therefore did not meet inclusion criteria

GFD: gluten-free diet; FODMAP: fermentable oligo-, di-, monosaccharides and polyols

Supplementary Table S2. Risk of bias assessment

Paper	Domain-specific risk of bias assessment							Overall risk of bias judgement
**Randomised controlled trials***								
	**ROB arising from the randomization process**	**ROB due to deviations from the intended interventions (assignment to intervention)**	**ROB due to deviations from the intended interventions (adhering to intervention)**	**ROB due to missing outcome data**	**ROB in measurement of the outcome**	**ROB due to selection of the reported result**		**Overall risk of bias judgement**
van Megen et al. 2022[25]	Low risk	High risk	High risk	Some concerns	Some concerns	Low risk		High risk
Roncoroni et al. 2018 [26]	Low-risk	High risk	High risk	Some concerns	Low risk	Low risk		High risk
Non-randomised interventional studies*								
	**Bias due to confounding**	**Bias in selection of participants into the study**	**Bias in classification of interventions**	**Bias due to deviations from intended interventions**	**Bias due to missing data**	**Bias in measurement of outcomes**	**Bias in selection of the reported result**	**Overall risk of bias judgement**
Testa et al. 2018 [27]	Critical risk	Low risk	Low risk	Moderate risk	Serious risk	Serious risk	Moderate risk	Critical risk
Trott et al. 2021 [28]	Critical risk	Serious risk	Low risk	Moderate risk	Low risk	Serious risk	Moderate risk	Critical risk
Observational studies**								
	**Selection score**		**Comparability score**		**Outcome score**			**Overall risk of bias judgement**
Cyrkot et al. 2021 [30]	2/3 points		1/2 points		3/3 points			Medium risk of bias (total 6/8 points)
Roncoroni et al. 2018 [29]	3/4 points		0/2 points		2/3 points			High risk of bias (total 5/9 points)

** Risk of bias evaluated using the Cochrane RoB 2.0 tool*

*** Risk of bias evaluated using the ROBINS-I tool*

**** Risk of bias evaluated using the Newcastle-Ottawa Quality Assessment scales for cross-sectional and cohort studies*
